# Colorectal cancer derived organotypic spheroids maintain essential tissue characteristics but adapt their metabolism in culture

**DOI:** 10.1186/1477-5956-12-39

**Published:** 2014-07-11

**Authors:** Uros Rajcevic, Jaco C Knol, Sander Piersma, Sébastien Bougnaud, Fred Fack, Eirik Sundlisaeter, Karl Søndenaa, Reidar Myklebust, Thang V Pham, Simone P Niclou, Connie R Jiménez

**Affiliations:** 1 NorLux Neuro-Oncology Laboratory, Department of Oncology, CRP-Santé, Luxembourg, Luxembourg; 2 OncoProteomics Laboratory, Department of Medical Oncology, VUmc-Cancer Center Amsterdam, VU University Medical Center, De Boelelaan 1117, 1081 HV Amsterdam, the Netherlands; 3 Department of Research and Development, Blood Transfusion Center of Slovenia, Ljubljana, Slovenia; 4 Department of Biomedicine, University of Bergen, Bergen, Norway; 5 Haukeland University Hospital, Bergen, Norway; 6 Department of Surgery, Haraldsplass Deaconal Hospital, University of Bergen, Bergen, Norway; 7 Institute of Pathology, Faculty of Medicine, University of Ljubljana, Ljubljana, Slovenia

**Keywords:** Colorectal cancer, Tissue spheroids, Proteomics, Mass spectrometry, Cancer stem cells, Bioinformatics, Cancer biomarkers, Label-free proteomics, 3D cell cultures

## Abstract

**Background:**

Organotypic tumor spheroids, a 3D *in vitro* model derived from patient tumor material, preserve tissue heterogeneity and retain structural tissue elements, thus replicating the *in vivo* tumor more closely than commonly used 2D and 3D cell line models. Such structures harbour tumorigenic cells, as revealed by xenograft implantation studies in animal models and maintain the genetic makeup of the original tumor material. The aim of our work was a morphological and proteomic characterization of organotypic spheroids derived from colorectal cancer tissue in order to get insight into their composition and associated biology.

**Results:**

Morphological analysis showed that spheroids were of about 250 μm in size and varied in structure, while the spheroid cells differed in shape and size and were tightly packed together by desmosomes and tight junctions. Our proteomic data revealed significant alterations in protein expression in organotypic tumor spheroids cultured as primary explants compared to primary colorectal cancer tissue. Components underlying cellular and tissue architecture were changed; nuclear DNA/ chromatin maintenance systems were up-regulated, whereas various mitochondrial components were down-regulated in spheroids. Most interestingly, the mesenchymal cells appear to be substantial component in such cellular assemblies. Thus the observed changes may partly occur in this cellular compartment. Finally, in the proteomics analysis stem cell-like characteristics were observed within the spheroid cellular assembly, reflected by accumulation of Alcam, Ctnnb1, Aldh1, Gpx2, and CD166. These findings were underlined by IHC analysis of Ctnnb1, CD24 and CD44, therefore warranting closer investigation of the tumorigenic compartment in this 3D culture model for tumor tissue.

**Conclusions:**

Our analysis of organotypic CRC tumor spheroids has identified biological processes associated with a mixture of cell types and states, including protein markers for mesenchymal and stem-like cells. This 3D tumor model in which tumor heterogeneity is preserved may represent an advantageous model system to investigate novel therapeutic approaches.

## Background

Preclinical cancer research has thrived on *in vitro* culturing systems. However, it has become increasingly clear that long term monolayer cultures used to study molecular and biological aspects of tumor cells, as well as their response to therapeutic agents, have lost a great many features exhibited by *in vivo* tumors. In cell monolayers, the architecture of a tumor tissue and the complex connections and interactions of its constituent cell types and matrix molecules are largely lost, intratumoral differences in nutrition, oxygenation, and drug penetration are abrogated, and growth on a plastic substratum can induce significant changes in cellular genotype and phenotype.

In contrast, three-dimensional cell culture systems have biological characteristics which are more closely related to those of *in vivo* tissue [1-4]. Spherical, multicellular aggregates called spheres can be grown from established cell lines or dissociated tissue cells in serum-free medium containing appropriate growth factors [5-9]. In recent years, sphere growth has become the basis of clonogenicity assays used to score for cancer stem cell-like cells (CSC). The latter are thought not only to underlie spheroid growth *in vitro*, but also to fuel tumor growth (and recurrence) *in vivo*, producing a similarly heterogeneous progeny of more differentiated cell types in both cases. Self-renewing at a relatively slow rate, CSCs are assumed to maintain an undifferentiated, damage-, apoptosis- and drug-resistant compartment of tumor-initiating cells. Thus, in contrast to their differentiated progeny, CSCs are clonogenic *in vitro* and tumorigenic *in vivo*, and therefore appear to be the main culprits in cancer biology. However, stem cell (−like) behavior may be a dynamic rather than a static property, *i.e.* a feature that can be adopted by more differentiated cells through interactions with the stromal environment [10] and/or through epithelial-mesenchymal transition (EMT) processes [11]. The Lgr5-positive crypt base columnar cell was suggested to represent the stem cell of the small intestine and colon [12]. It has also been described for normal colon biology that the transfer of stem cells between the border and central regions of crypts, endows all Lgr5+ colon stem cells with long-term self-renewal potential through which a heterogeneous cell population is able to function long term as a single stem-cell pool [13]. In mice, the Lgr5-expressing colon stem cells depend on stem cell niche signals provided by a specialized daughter cell, the (CD24+)Paneth cell [14,15]. The colon CSC compartment maintenance may depend on equivalent niche signals.

Detailed cellular and molecular characterization of CSCs, including their specific protein expression profiles, may lead to a better understanding of tumor biology, and provide novel biomarkers and targets for therapeutic procedures. Different markers have been proposed for the identification of colorectal CSCs, including CD133 (Prominin-1), CD166 (Alcam), CD44, EpCAM, CD24, CD29 (Integrin beta-1) and others [16] as well as the Lgr5 protein found in normal intestinal stem cells [16-21]. In addition to these cell surface markers, the use of intracellular markers involved in epithelial development and differentiation has been proposed to identify CSCs [22-24]. For example, Wnt signaling cascades, where nuclear b-catenin is one of the key players (reviewed in [16]), have important roles in the homeostatic self renewal of the intestine as well as in colorectal carcinogenesis. Thus, their role in colorectal cancer ‘stemness’ has received particular attention [25-27].

Spheroids can not only be generated from (dissociated) cultured cells, but also directly from tissue biopsy fragments in an organ culture type of fashion [28-32]. Importantly, these “tumor tissue spheroids” preserve tissue characteristics of the original tumor *in situ* that are not only lost in monolayer cultures of cell lines, but also in spheroids derived from dissociated tissue cells. Specifically, tissue elements such as extracellular matrix, capillaries, connective tissue, and immune cells can be demonstrated in such ‘organotypic’ spheroids as well as tumor cell heterogeneity [28]. These tissue spheroids still preserve tumor-initiating potential as demonstrated by xenografting experiments in animal models, where they generated stem-like tumor phenotype [33]. Moreover, in case of glioblastoma, the spheroids themselves display some features of stemness evident in the expression of certain markers and cell growth patterns [33]. In addition to that, in colorectal cancer research tissue culture protocols were reported that allow the isolation and propagation of tumor-derived 3D-spheroid/organoid cultures that are enriched in CSCs [19,23,34,35]. It is also well known that genomic profiles of long term cell cultures frequently deviate from parental tumor profiles, whereas spheroids are genetically more stable and representative of the original tumor [36]. Therefore, with a complexity that is lower than that of the original tumor, but higher than that of other *in vitro* culture systems, organotypic tissue spheroids could be used to maintain and study tumor-like tissue and test therapeutic agents *in vitro* in a more relevant setting. Recently, successful 3D culture system was also described that allows long-term expansion of adult small intestine, stomach and liver cells without the need of a mesenchymal niche, while preserving the characteristics of the original adult epithelium [37-39] using the Wnt agonist RSPO1 in culture medium [40-43].

Here, we have established colorectal carcinoma (CRC) spheroid cultures from patient resection material. These spheroids develop into an outer layer of epithelial-like cells and an inner core of mesenchymal-like cells. To characterize the spheroid-specific protein repertoire and to assess the similarities between spheroids and biopsies at the protein level, we have performed an in-depth differential proteomics analysis of CRC tissue spheroids versus the original tissue resection material from which they were derived. To the best of our knowledge, this is the first in-depth proteomics characterization of primary tumor (tissue) spheroids that includes identification and relative quantification of a significant set of proteins on a high-end mass spectrometric platform. Biological network and pathway analysis of proteins that were differential between tumor spheroids and their parental tumors indicate that nuclear (chromatin, DNA, and RNA regulatory) proteins, cytoskeletal and cell-cell/cell-substratum interaction proteins, are up-regulated, whereas mitochondrial and metabolic processes are clearly down-regulated. Although harboring a mixed population of cells and their associated cellular processes, organotypic spheroids show elevation of a number of putative markers associated with CSCs, such as oxidative stress-induced proteins like Aldh1 and Gpx2, and the cell surface marker CD166 (Alcam1) and b-catenin. Therefore this system may prove relevant for drug testing and yield novel drug targets and markers for further testing in pre-clinical animal models.

## Results and discussion

### Tumor spheroid formation

**Figure 1:**
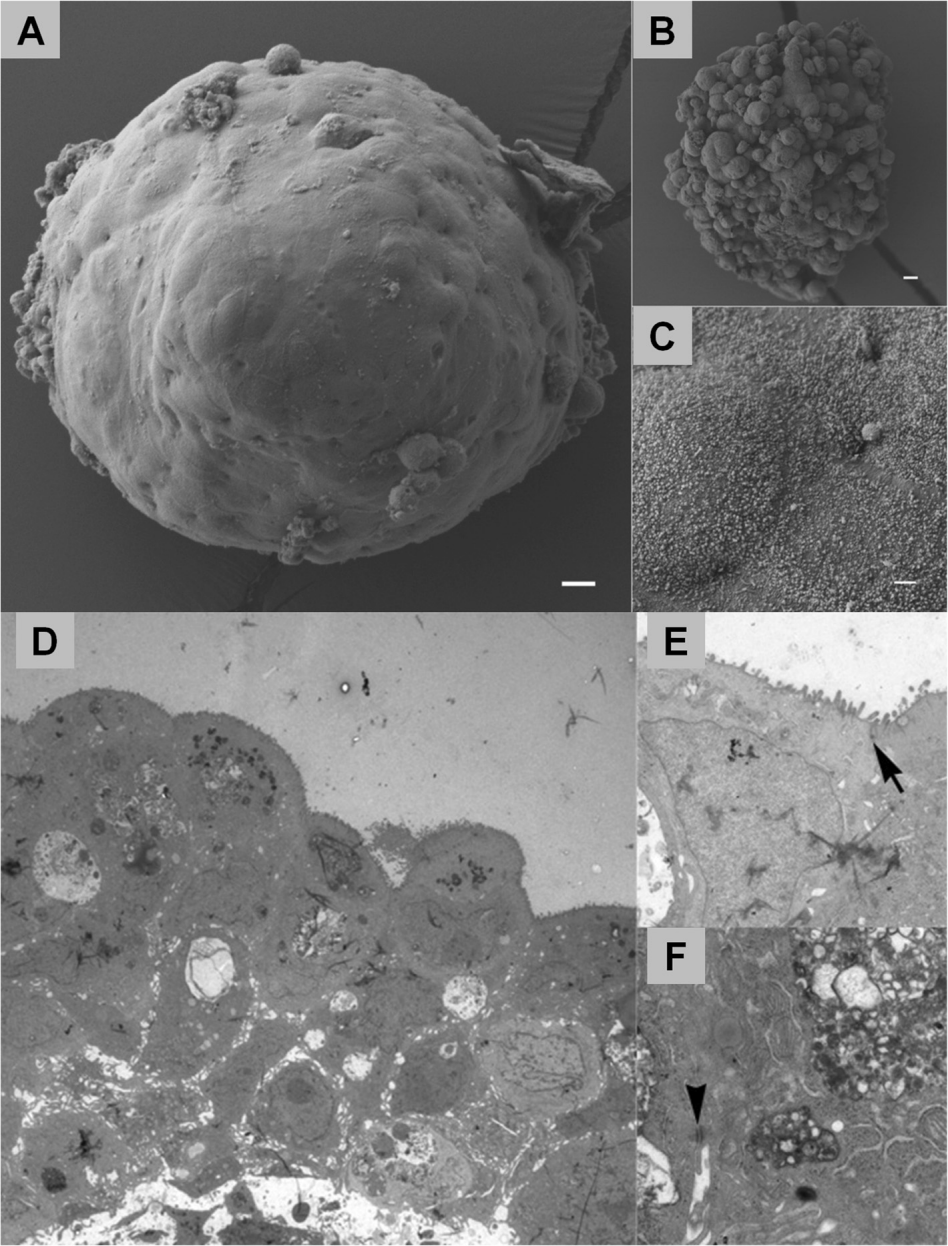
**Electron microscopy showing ultrastructure of the colorectal cancer spheroids under investigation.** Scanning electron micrograph of spheroids grown from different tumors **(A, B)** showing variation in surface architecture. Scale bars = 10 μm. The presence of microvilli was revealed on greater magnification **(C)**. Scale bar = 1 μm. **(D-F)** Transmission electron microscopy. Transmission electron micrograph from a spheroid after 10 days of culture **(D)**. Note the nuclear pleomorphism indicating tumor cell heterogeneity, and the presence of microvilli (small arrow) at the apical surface of peripherally located cells **(E)**. Cells were closely interrelated; tight junctions (large arrow) in **(E)** and desmosome junctions (arrowhead) were observed between neighboring cells **(F)**. Magnification: D; 2000×, E; 12000× and F; 15000×.

We have generated tumor tissue spheroids from human colorectal tumor resection material. Of 12 colon adenocarcinomas studied, 11 formed spheroids, usually after 2–5 days in culture. In contrast, normal colonic mucosa always failed to form spheroids in culture. The number of spheroids formed from each tumor depended on the amount of biopsy material, but generally the number of spheroids was over 30. Only tissue fragments that formed spherical aggregates within 5 days in culture were used for further study. Spheroid size varied between 150–350 μm in diameter and average size was about 250 μm (Figure 1A). The spheroids from three (Table 1; highlighted CC9, 11, 12) colon adenocarcinomas that were further analyzed remained stable in size over time in a 20-day culturing period. Also, when comparing spheroids from different patients, no significant difference in spheroid size could be discerned (Additional file 1: Figure S1A).

**Table 1:** Clinical patient data and corresponding colorectal carcinoma samples grown as spheroids in vitro

**Patient #**	**Gender**	**Age**	**Tumor localization**^**a**^	**TNM Stage (I-IV)**	**Spheroid formation**
**1**	M	65	Distal	III	Yes
**2**	F	48	Distal	II	Yes
**3**	F	73	Distal	II	Yes
**4**	M	83	Distal	III	Yes
**5**	F	81	Proximal	III	Yes
**6**	M	69	Proximal	II	No
**7**	M	74	Distal	III	Yes
**8**	F	82	Distal	II	Yes
**9**	**M**	**52**	**Proximal**	**III**	**Yes**
10	M	85	Distal	II	Yes
**11**	**F**	**56**	**Proximal**	**III**	**Yes**
**12**	**F**	**47**	**Distal**	**III**	**Yes**

Samples of patients 9, 11, 12 (highlighted in red) were used for proteomics.

### Microscopical and immunohistochemical characterization of tumor spheroids

Scanning electron microscopy of tumor tissue spheroids revealed a somewhat variable spheroid structure, ranging from a largely smooth surface (Figure 1A) to a more blebby appearance (Figure 1B). This variation was observed even for spheroids derived from the same tumor. Also, structures that seemed to be the remnants of a vessel were present on the surface of one spheroid (not shown). On a higher SEM magnification (Figure 1C), small scale ‘brush’ structures can be seen on the spheroid surface. In transmission electron microscopical images, these structures were observed at the surface of peripheral cells (Figure 1D), with a rather irregular shape and thickness (see higher magnification in Figure 1E). This suggests that these cells preserve, to some extent, a polarized epithelial-like character. TEM revealed a considerable variation in shape and size of spheroid cells, which were closely interconnected and joined by desmosomes (1E large arrow) and tight junctions (Figure 1F, arrowhead). Characteristically, the nuclei were hyperchromatic and large, and the cytoplasm showed unusual high numbers of vacuoles and structures similar to lysosomes (Figure 1D).

**Figure 2:**
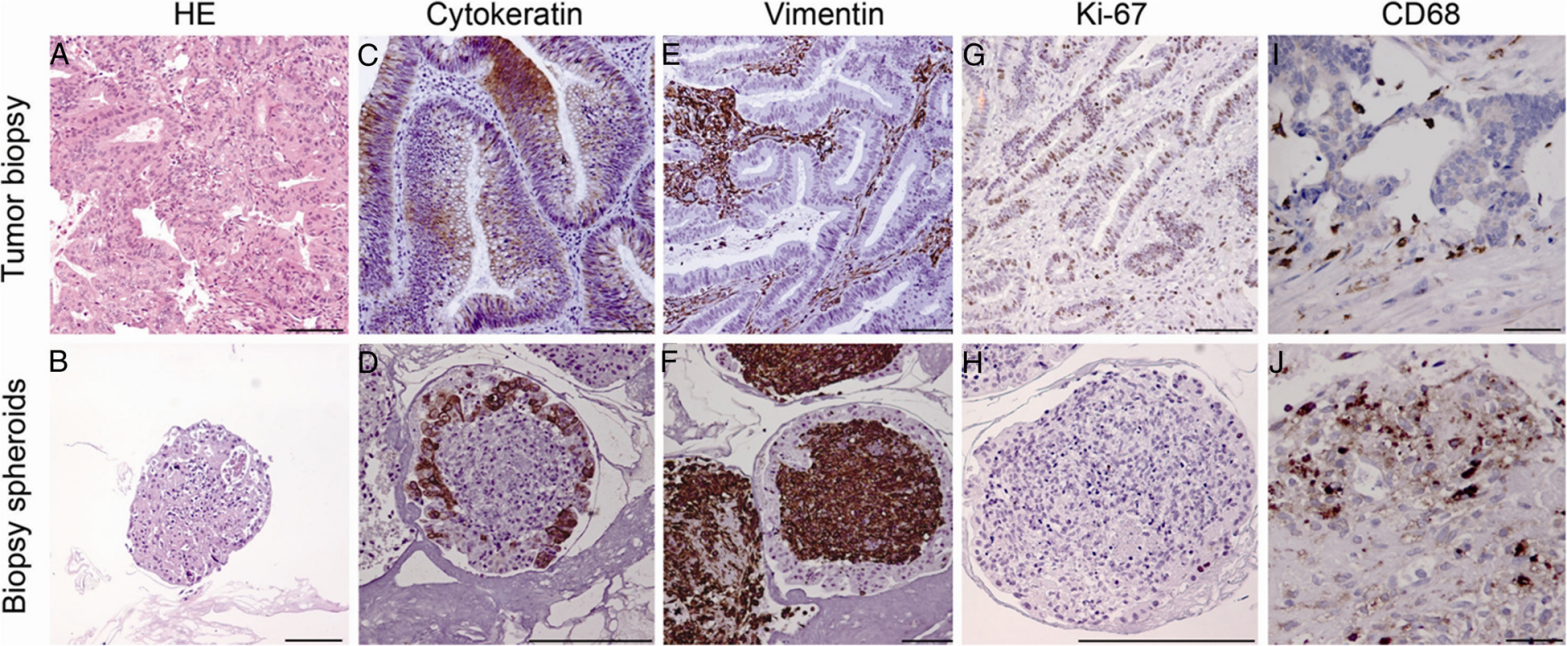
**Immunohistochemical staining of tumor biopsy and biopsy spheroid sections.** HE, haematoxylin & eosin staining. Cytokeratin, staining with anti-CK20 (Krt20) antibody. Vimentin, staining with anti-vimentin (Vim) antibody. Ki-67, staining with anti-Ki-67 (Mki67) antibody. CD68, staining with anti-CD68 antibody. See Materials and Methods for details.

Figure 2 shows H&E stainings as well as immunohistochemical characterization of CRC tissue biopsies and biopsy-derived spheroids. Generally, as observed in TEM, in H&E-stained sections spheroid cells were tightly packed together (Figure 2A, B). Interestingly spheroids often resumed a 3D structure that was reminiscent of the intestinal tissue organization with an outer layer of cells, one to five cells thick that seemed to be of an epithelial nature. The epithelial character was confirmed by staining for cytokeratin 20 (CK20/Krt20), an established marker for colonic epithelial cells (Figure 2C, D). In contrast, CK20 staining was undetectable in the central core of spheroids. The converse was true for the mesenchymal marker, vimentin (Vim), which was detected in the central core, but not in the epithelial-like layer (Figure 2E, F). To assess proliferative activity, spheroids were stained for the proliferation marker, Ki-67/Mki67 (Figure 2G, H). Whereas the proliferation index was calculated to be 51.6% for tumor tissue, it was only 5.5% within spheroids indicating that 10-day-old organotypic tumor spheroids contain few cycling cells (Additional file 1: Figure S1B), which is consistent with the absence of spheroid growth over time. Figure 2I, J also shows an example of tumor infiltrating immune cells, as revealed by staining for the macrophage-associated CD68 marker, that could still be found in spheroids (Figure 2J, suggesting that the organotypic spheroid culture model still retains non-neoplastic cells of the tumor microenvironment and thus approximates the *in vivo* situation more than monolayer cultures or monoclonal spheroids do. Given the cellular ratio of epithelial-like versus mesenchymal-like cells, it is to be expected that whole-spheroid analyses will be dominated by the latter.

### Differential proteomics of CRC tumor tissue and CRC tissue spheroids

To assess relative differences in protein expression between CRC tumor tissue and organotypic spheroids derived thereof, lysates of tumor tissue as well as corresponding spheroids were prepared with material from three patients. We set out to fractionate similar protein amounts from each lysate on an SDS-PAGE gel (Additional file 2: Figure S2), process individual gel slices through in-gel tryptic digestion, and analyze the resultant tryptic peptide mixtures using label-free nanoLC-MS/MS. Subsequently, we performed database searching and spectral counting as described before [44,45] to discern relative differences in protein expression between different samples.

**Figure 3:**
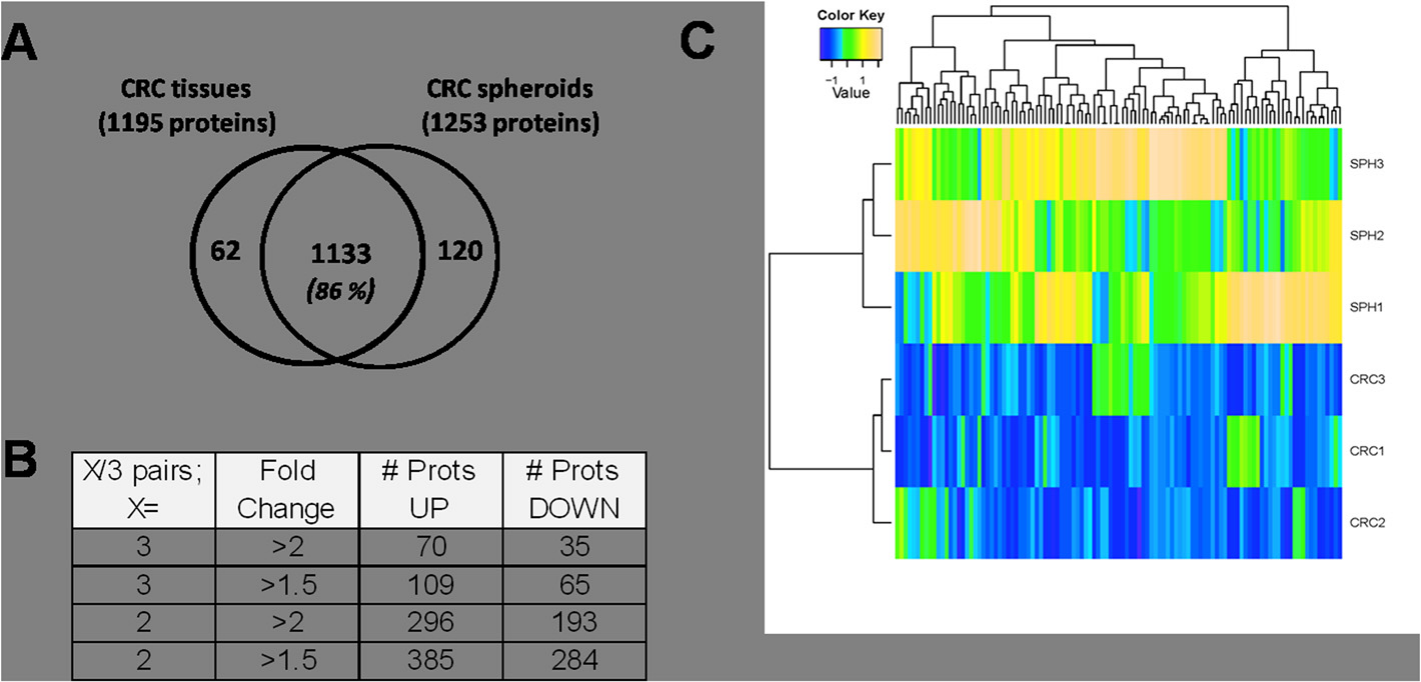
**Summary of the protein expression data in colorectal tumors vs corresponding spheroids. (A)** Venn diagrams illustrating overlapping sets of unique proteins identified in CRC tissues and CRC spheroids derived thereof. **(B)** Tables denoting the number of differential proteins, respectively, with different criteria for filtering. Abbreviations: CRC, colorectal tumor tissue; SPH, CRC spheroids; FC, fold change; Sp. Count separated, number of proteins from the third column (p < 0.05 & FC > 2) that, in addition, exhibit spectral count separation: the lowest spectral count in one group is higher than the highest spectral count in the other group. **(C)** Heat map view of a supervised cluster analysis using differential proteins from the comparison CRC tissue and spheroid proteomes (174 proteins with 1.5 fold change in all 3 pairs). The protein abundances were normalized to zero mean and unit variance for each protein. Two clusters are formed for the CRC tissue lysate and spheroid groups. CRC, colorectal cancer; tissue sample; SPH, spheroid sample.

Our analysis yielded a dataset of 1366 IPI database entries (1315 unique proteins) that were identified across all samples (see Materials and Methods for identification criteria). In each group, the overlap of protein identification across all three biological samples was over 50% (Additional file 3: Figure S3). The majority (1133) of the 1315 proteins (86%) was found in both tumor tissue and spheroids, whereas a minority was specific to either sample type (Figure 3A). This suggests a high similarity between spheroids and parental tumor tissue. Differential details of the dataset are shown in Figure 3B. A subset of 174 proteins exhibited > 1.5-fold change in each of the pairs, and 105 proteins of those additionally exhibited a more than twofold difference between tumor tissues and spheroids (70 up-regulated, 35 down-regulated) (Figure 3B). A supervised cluster analysis using these 174 differential proteins is shown in a heat map view in Figure 3C. Two main clusters are discerned, corresponding to the CRC tissue samples and the tissue spheroid samples, respectively. The up-regulated proteins contained a subset of 26 acute phase, coagulation, and complement cascade proteins (Additional file 4: Table S1) that are at least partly introduced by the medium used for spheroid culturing, and were therefore not subjected to further analysis. The remainder of the differentially expressed proteins are listed in Tables 2A, 2B, 3A and 3B. The whole dataset is provided in Additional file 5: Table S2.

**Figure 4:**
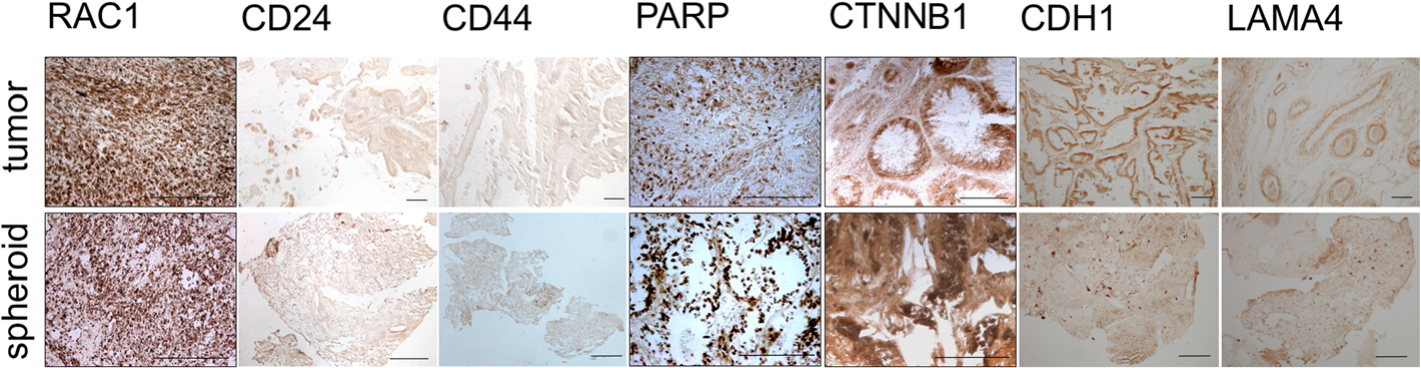
**Immunohistochemical staining of CRC biopsies and corresponding spheroids.** The section shows mainly the internal, mesenchymal part of the spheroid. Scale bar is 100 μm for all images.

Our proteomics data indicate an accumulation in mesenchymal-like cells (as deduced from the twofold up-regulation of the mesenchymal marker vimentin) and a decrease in epithelial-like cells (as deduced from the overall down-regulation of several markers: cytokeratin 20, mucin 2 (Muc2), and vilin 1 (Vil1)) in spheroids as detailed in Additional file 5: Table S2). Epithelial markers such as E-cadherin (Cdh1) see also (Figure 4) and Epcam appear to be relatively unchanged. Loss of the former is associated with tumor progression and invasion [46], whereas the latter has been associated with the colonic cancer stem cell (Co-CSC) compartment [19]. The far majority of the regulated proteins reported in Table 1A (68 of 70) have a ratio of ~ 3 and higher while vimentin is upregulated in spheroids only with a Fc of 1.9 (Table 2B, 3B). Therefore, the differential expression of most proteins in our top list cannot be solely explained by the epithelial/stroma ratio.

### Protein network analysis for differential proteins

**Figure 5:**
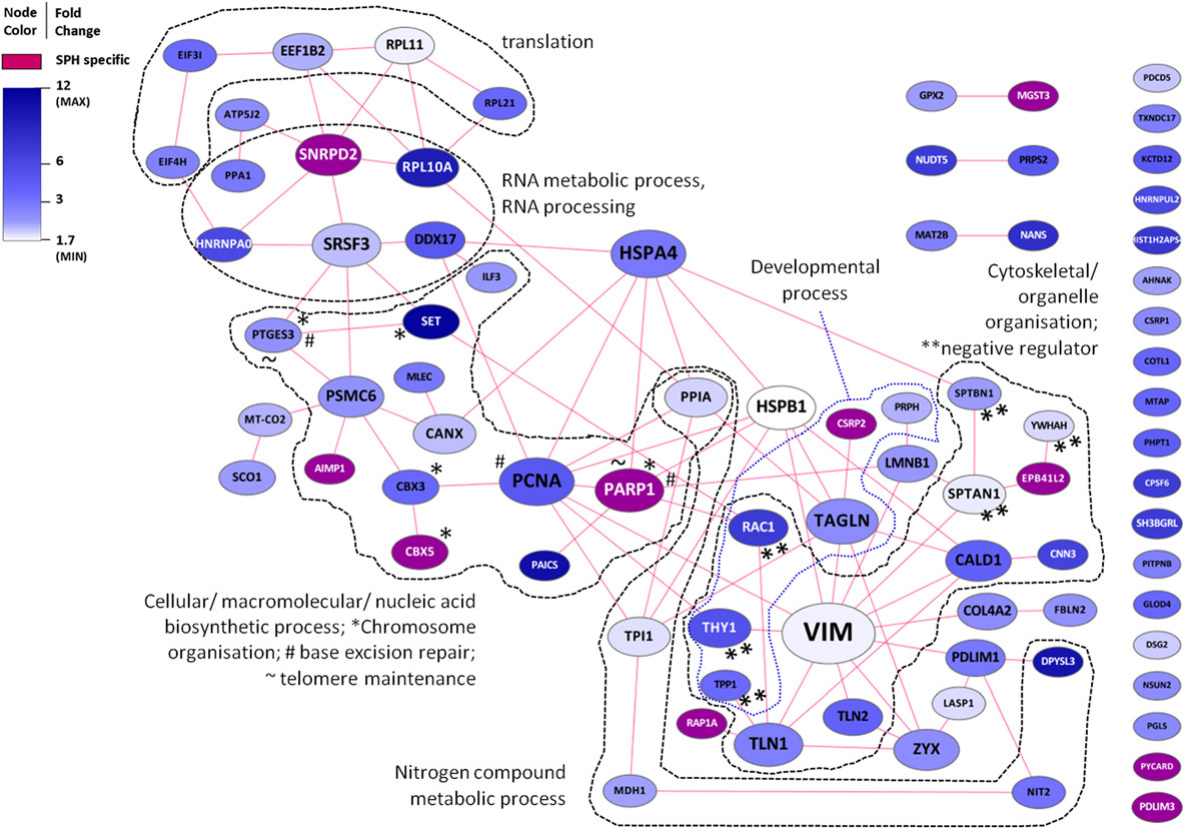
**Network of up-regulated proteins (referenced by gene symbols) in all CRC-SPH pairs.** Proteins up-regulated with a fold change > 1.5 in all three pairs of spheroids (SPH) versus their parent colorectal carcinoma tissue (CRC) were pre-screened with the BINGO gene ontology tool, and inflammatory/acute phase-related proteins were deleted from the list. The remaining proteins were uploaded to STRING, and the returned protein-protein link data were used to generate and annotate a protein network in Cytoscape. Nodes and their labels were sized according to the number of edges connected to them. Node color was made dependent on the *overall* fold change in all three CRC-SPH pairs: nodes exhibiting a defined fold change were colored along a white-to-blue gradient (minimum and maximum values indicated), whereas nodes only detected in SPH were colored purple, see color legend.

**Figure 6:**
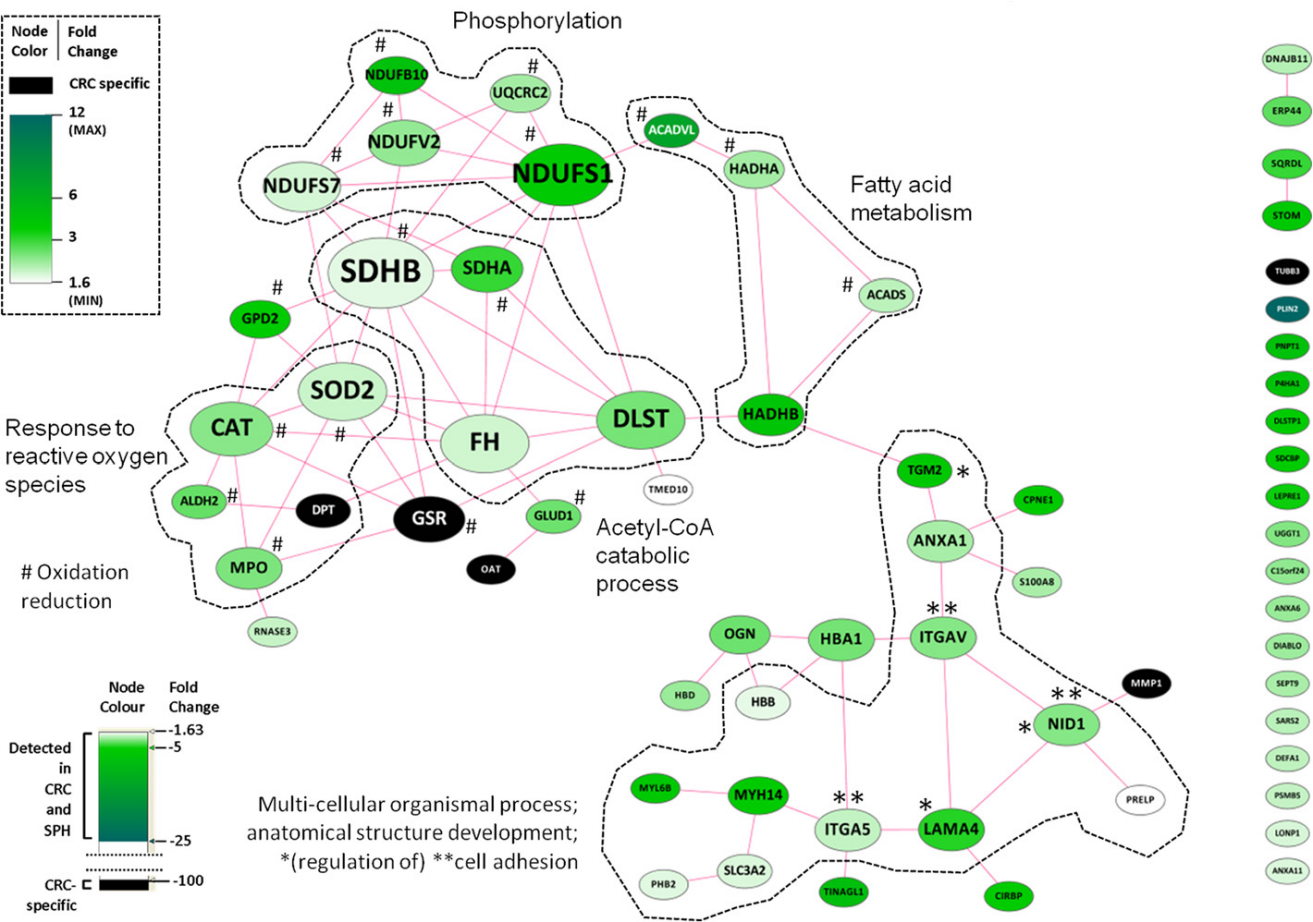
**Network of down-regulated proteins (referenced by gene symbols) in all CRC-SPH pairs.** Proteins down-regulated with a fold change > 1.5 in all three pairs of spheroids (SPH) versus their parent colorectal carcinoma tissue (CRC) were uploaded to STRING, and the returned protein-protein link data were used to generate and annotate a protein network in Cytoscape. Nodes and their labels were sized according to the number of edges connected to them. Node color was made dependent on the *overall* fold change in all three CRC-SPH pairs: nodes exhibiting a defined fold change were colored along a white-to-green gradient (minimum and maximum value indicated *for connected nodes*), whereas nodes only detected in SPH were colored black, see color legend. The unconnected PLIN2 node has a fold change of 24, but was given the color of the top of the gradient.

To obtain a first impression of the type (s) of regulated proteins that are functionally interconnected in our dataset, we fed the lists of Tables 2 and 3 to the web-based STRING tool [47], which mines known and predicted protein interactions. These networks were imported and visualized in Cytoscape [48] (Figures 5 and 6), and associated biological functions were explored using the BINGO plugin for Cytoscape [49]) (Additional file 6: Table S3). In the network of proteins up-regulated in spheroids (Figure 5), one can discern [1] a set of proteins involved in translation (Rpl11, Rpl21, Eif3i, Eif4h, Eef1b2); [2] a characteristic RNA splicing factor network involved in binding, metabolism, and processing of nuclear RNA (e.g., Hnrnpa0, Srsf3, Ddx17); [3] a layer of interacting proteins involved in chromatin anchoring and epigenetic/transcriptional regulation (Cbx3, Cbx5, Set, Ilf3), DNA replication (Set, Pcna), or DNA repair (Parp1, Pcna); [4] two interconnecting heat shock proteins (HSPA4 and HSPB1) [5] a set of components involved in cytoskeletal organization, organelle organization, cell-cell interactions, and signal transmission, including Vim, talins (Tln1, Tln2), the anti-mitogenic protein Rap1A, Rac1, and Zyx; [6] tissue development proteins (including Tagln, Csrp2, Rac1); and [7] a set of proteins involved in nitrogen compound metabolism (PpIa, Tpi1, Mdh1, Nit2, Dpysl3)).

At a lower stringency level, by including proteins up-regulated in spheroids in at least two out of three sample pairs (Additional file 7: Table S4), further support is obtained for an increase in: RNA splicing factors and proteins involved in DNA replication, nucleosome reorganization and transcription (Mcm3, Mcm7, Ssrp1), several additional nuclear lamina/scaffold proteins (Lmnb1, Emd, Matr3, Numa1, Ncl), proteins involved in shuttling between the cell cortex/focal adhesions and the nucleus (δ-catenin (Ctnnd1), Lpp, Ahnak), and the central Wnt pathway effector β-catenin (Ctnnb1). The latter protein has been strongly implicated in ‘stemness’ of both normal and cancer stem cells of the colon. Among the set of up-regulated proteins in ≥ 2/3 pairs (Additional file 7: Table S4), we also discern proteins involved in reducing cellular stress (Hsph1, Gsto1, Gstm1, Gpx2, Aldh1a1). The latter two proteins have been recently associated with, and proposed as markers for, colonic CSCs [23].

Among the (core of) down-regulated proteins (Figure 6), there is a preponderance of interconnected mitochondrial enzymes involved in phosphorylation, fatty acid metabolism, acetyl-CoA catabolism, and responses to reactive oxygen species. This correlates with a reduced metabolic rate, in particular a reduction in cell proliferation and in oxidative phosphorylation, which is in agreement with the slow cycling activity and the reduced oxygen tension present in the spheroids. Some of the down-regulated proteins are involved in reducing oxidative stress (Cat, Sod2, Gsr), whereas others may contribute to such stress (e.g. mitochondrial respiratory chain components) or apoptosis (Tgm2). Overall mitochondrial activity seems to be significantly reduced, although, interestingly, and against the trend, both cytochrome c oxidize (Mt-co2) and one of its biosynthetic chaperones (Sco1) are up-regulated. In addition, a set of proteins associated with anatomical structure development involving cell-substratum interactions and extracellular matrix composition/modulation are down-regulated (Lama4, Myl6b, Itga5, Phb2, Itgav, Tgm2, Anxa1, Nid1, Hbb, Prelp). In all, the nature of the down-regulated proteins suggests a reduction of mitochondrial activity and a change in the cell-substratum interface, going from CRC tumor tissue to spheroids *in vitro*. Furthermore, the down-regulation of the Mpo peroxidase seems to indicate a relative depletion of immune cells in spheroids.

### Immunohistochemistry of selected spheroid-enriched proteins

The expression of a number of proteins encountered in the above charts has been further characterized through immunohistochemical staining (Figure 4). These proteins include the nuclear protein Parp1 involved in DNA repair (Figure 4), and the nuclear and/or cytoplasmic Wnt pathway components β-catenin and Rac1 implicated in stemness (Figure 4). As expected these proteins could be confirmed both in spheroids and the tumor biopsies, further supporting the similarities between the samples. Similar expression of Cdh1 in tissue vs spheroids as found by proteomics was confirmed by IHC staining (Figure 4). The proposed markers of cell stemness CD44 and CD24 were also included in the IHC analysis and showed slight increase in spheroids (Figure 4). Finally, the down-regulation of the cell-cell-interaction protein LAMA4 in the spheroids as determined by proteomics was confirmed by IHC (Figure 4), possibly indicating a more fluid organization of ECM and cell-cell interactions in spheroids.

### Functional implications of tumor spheroid analyses

Being able to study tumorigenic cells in their native niche, but *ex vivo*, is invaluable for gleaning information about basic cancer biology and tumor cell resistance, as well as for early *in vitro* testing of new candidate anti-tumor drugs. Although monolayer (2D) cultures of established cell lines and spheroid (3D) cultures of both tumor cell lines and dissociated tumor tissue cells have provided us with useful information, these models are of monoclonal origin and have lost the complexity of a heterogeneous cancer tissue. Organotypic spheroids such as used here, which preserve multicellularity throughout the procedure and retain original tissue elements [28], more closely resemble the tissue *in vivo*. Such structures harbor tumor initiating cells, as revealed by xenograft implantation studies in animal models [33]. According to the CSC concept, such cells should exhibit characteristics linked with ordinary stem cells, such as slow cycling [50], enhanced DNA repair, chromatin maintenance and telomerase activity [51], resistance to (oxidative) stress and apoptosis [52-55], and drug detoxification and efflux (Additional file 5: Table S2) [50]. Here, we have performed an initial proteomic characterization of organotypic spheroids derived from CRC tissue, which, following *in vitro* culture, appear to be enriched in stem-like cells, while maintaining the cellular complexity of the parental tumor.The CRC tumor spheroids as grown here, exhibit at least a dichotomy of cell types: the outer surface is made up of epithelial-like cells, whereas the inner core contains substantial part of mesenchymal-like cells recapitulating the tissue of origin. The observed ultrastructural variability (Figure 1A and B) might be explained by a differential extent of (outer) extracellular matrix deposition/ remodeling, a process that according to our proteomics data is ongoing in spheroids relative to parental tumor tissue. Indeed, as the generation of spheroids from primary tissue will be accompanied by significant changes in cell and tissue architecture, it is not unexpected that various proteins associated with the extracellular matrix, the cell cortex, and the cellular cytoskeleton are found upregulated in spheroids (see Tables 2 and 3, Figures 5 and 6). Apart from these changes, we observe up-regulation of specific proteins associated with protein translation, RNA metabolism (RNP components), and DNA/chromatin/chromosome organization and maintenance, as well as down-regulation of a significant set of mitochondrial proteins.

Ki-67 staining suggests that very little proliferative activity occurs in spheroids. When we inspect our differential proteomics data on up-regulated proteins, we encounter the Pcna protein and the Mcm proteins, all classically implicated in DNA replication. However, the above molecules may serve more complex functions. Thus, Mcm proteins are stably loaded onto chromatin in the G1 phase, but require triggering of their helicase activity and ensuing replication to unload; following replication arrest, they remain bound indefinitely [56]. Pcna, a binding partner for a plethora of proteins, and subject to diverse post-translational modifications, has been dubbed the “maestro of the replication fork”, and is involved in multiple processes including, besides DNA replication, DNA repair, chromatin remodeling, sister-chromatid cohesion, and cell cycle control [57,58]. These proteins may accumulate in cell cycle arrested cells and thus, replication does not necessarily have to be the major differential DNA/chromatin-associated process when comparing CRC tumor tissue with the spheroids grown from that tissue.

Indeed, it seems that the nuclear proteins upregulated in spheroids form part of a larger array of proteins involved in chromatin regulation/maintenance as well as DNA repair. Cbx proteins have been classically associated with epigenetic repression of gene expression (binding to histone H3 in silenced chromatin), although they also seem to have a role in transcription [59,60]. Furthermore, they are known to connect chromatin to the nuclear lamina through the lamin B receptor [61]. In our dataset, in the lower stringency analysis, Cbx3 (also known as HP1γ) and Cbx5 (also known as HP1α) are both connected to the histone H3f3a variant (H3.3) which is not only linked with active gene transcription [62], but also with heterochromatin formation [63], and possibly lineage priming in mesenchymal stem cells [64]. Moreover, histones H2a (a nucleosome constituent) and HIST2H2BE Hist2h2be (both upregulated in two out of three spheroid samples) play an important role (may contribute to the formation of the functional antimicrobial barrier of the colonic epithelium) in epigenetic control of stem cells [65]. It remains however, to be analyzed whether the chromatin-modification reflects a changing stress response. Furthermore, the Set protein has been linked with inhibition of both HAT-dependent transcription [66] and DNA demethylation [67], thus enhancing gene silencing. Set is also an anti-apoptotic component involved in caspase-independent apoptosis. Another multifaceted protein that is upregulated in spheroids is the DNA repair protein Parp1 [68,69]. One of the additional roles of Parp1 is induction of repressed chromatin [70], and it has also been associated with telomere maintenance [71].

One of the nuclear spheroid proteins that warrants follow-up studies as a candidate prognostic and drug resistance biomarker in CRC is ILF3. ILF3 was also identified as spheroid enriched protein in our previous proteomics screen of single cell-derived CRC spheres [72]. This protein is a double-stranded RNA (dsRNA) binding protein that complexes with other proteins, dsRNAs, small noncoding RNAs, and mRNAs to regulate gene expression and stabilize mRNAs, and has an emerging function in DNA repair. In translational studies, ILF3 was part of a gene signature that can predict survival in lung cancer patients which is relevant for the selection of patients for adjuvant therapy [73]. In the latter study, ILF3 showed correlated mRNA and protein overexpression in lung cancer development and progression. In another study, ILF3 was part of a gene expression pattern for doxorubicin (Adriamycin) resistance where resistant breast tumors highly expressed this gene [74]. In all, it appears that CRC tissue spheroids harbor chromatin maintenance and DNA repair processes that are enhanced to some degree.

When inspecting the proteins that are down-regulated in spheroids versus CRC tumor tissue, one is immediately struck by the widespread down-regulation of mitochondrial proteins, including multiple respiratory chain components. Reprogramming of energy metabolism is an emerging hallmark of cancer cells [75], and tumor cell metabolism a relatively new focus of attention [76]. Metabolic reprogramming in response to hypoxia is associated with several well-known regulators implicated in cancer biology, including p53, PI3K, NFκB, HIF1, and Myc [77]. In this light it is of note that loss-of-function mutations of fumarate hydratase (Fh) and succinate dehydrogenase (Sdh) components (down-regulated in our study, Figure 6) have been associated with certain tumors, and that the resulting accumulation of fumarate or succinate can counter the destruction of Hif1a [76]. Also, reduced conversion of acetyl-CoA (see Figure 6) may make this key intermediary available for histone acetyl transferases that mark the genome epigenetically [78]. Shunting glucose into the pentose phosphate pathway instead of mitochondrial oxidative phosphorylation allows cells to generate NADPH, a key molecule in anti-oxidant defense [76]. The concomitant down-regulation of anti-oxidant proteins such as Sod2 and Gsr (as well as peroxisomal Cat) may be overcome by sufficient dampening of mitochondrial activity, as well as by the up-regulation of oxidative stress-reducing proteins such as Mgst3 [79], and Gpx2 [24]. Other proteins that we found to be differential may also contribute to the detoxification of harmful compounds, such as Nit2 [80], Cbr1, Aldh1a1, as well as other up-regulated dehydrogenases in our dataset (Additional file 5: Table S2) [81,82]. In relation to these defense mechanisms, cell survival through inhibition of apoptosis might also occur. Here, we see a somewhat mixed picture: whereas pro-apoptotic molecules such as Pycard, and Pdcd5 exhibit elevated levels, anti-apoptotic proteins such as Set and Hspb1 are up-regulated, in addition to the down-regulation of death-inducing proteins such as Diablo and Tgm2. The latter protein is normally squelched by Gstp1 [83,84], a protein which is slightly up-regulated in our dataset. Mitochondrial alterations may also affect the role these organelles normally play in cellular apoptosis [85]. Together, our observations suggest that CRC tumor spheroids try to ward off various forms of cellular stress, including hypoxia.

We have detected increased levels of β-catenin (Ctnnb1) a key component of the classical Wnt pathway [86,87], in our spheroids (Additional file 7: Table S4). Both Parp1 and Xrcc6 (also known as Ku70), proteins found in our dataset to be up-regulated in spheroids, can bind to the Tcf4/β-catenin transcription factor complex central to Wnt signaling [88,89]. Since β-catenin has turned out to be a hallmark protein defining “Wnt activity” and “stemness” in colorectal stem cells and CSCs [10,86], one would surmise that tumor spheroids, harboring tumor-initiating cells, contain cells that display such activity. However, “Wnt activity” is determined by nuclear localization of the β-catenin protein [10,86], the extent of which we cannot assess from our data.

In recent years, attempts have been made to characterize (and sort) tumorigenic, colorectal CSCs through cell surface markers such as CD133 (Prom1), CD166 (Alcam), CD44, EpCAM, CD29 (Itgb1), and CD24 [17,18,21,22]. Especially the CD133 marker has received much focus [18,19], but has become somewhat controversial [90], and concerns have been raised regarding the antibody used to detect the CD133 antigen [91]. Of the above, in our proteomics study, we have only detected Alcam as a differential protein in 2 of 3 tissue-spheroid pairs, in addition to CD44, which was only differential in one pair. We did, however, also observe significantly differential levels for the intracellular Aldh1a1 and Gpx2 proteins which were recently associated with the colorectal CSC compartment [23,24]. In line with the above, the IHC analyses of putative colorectal tumor stem cell markers showed increased expression of Ctnnb1, CD24 and CD44 in spheroids vs tumors by IHC (Figure 4) thus indicating the presence of a cancer stem cell compartment.

## Conclusions

In sum, collectively our data reveal significant similarities between organotypic CRC spheroids and primary CRC tumor tissue. The differences detected at the protein level include components underlying cellular and tissue architecture; an upregulation of nuclear DNA/ chromatin maintenance systems and a down-regulation of various mitochondrial components in spheroids. In view of the substantial content of mesenchymal cells in such cellular assemblies, these changes may largely occur in this compartment. We have also observed some stem cell-like characteristics within the mix of different cell types and cell states present in spheroids, including Alcam, Aldh1, Gpx2, Ctnnb1, CD24, CD44, and CD166 warranting closer investigation of the tumorigenic compartment in this 3D culture model for tumor tissue as well as the use for *in vitro* drug testing.

## Patients, materials and methods

### Patient tumor material

Tumor fragments were obtained after surgery from 12 patients with colorectal carcinoma (Table 1). All the tumors were classified histologically as adenocarcinomas by a pathologist. Consent was given by the patients for the use of the tissue for experimental purposes, and investigations were approved by the ethical committee of the Haukeland University Hospital, Bergen, Norway. The specimen obtained at surgery were immediately transferred aseptically to a test tube containing growth medium: Dulbecco’s modified Eagle’s medium supplemented with 10% fetal calf serum (FCS), four times the prescribed concentration of nonessential amino acids, 4 mM L-glutamine, penicillin (100 IU/ml), streptomycin (100 μg/ml) and Fungin™ (10 μg/ml) (Bio Whittaker, Verviers, Belgium).

### Cell culture and spheroid growth

Specimen selected for three-dimensional (spheroid) culture were cut, minced, and incubated in 75-cm^2^ agar-coated tissue culture flasks (Nunc, Roskilde, Denmark) with 30 ml growth medium (described above). The growth medium was changed every second day. After 2–5 days of culture in a standard tissue culture incubator (5% CO_2_, 100% air humidity), the tissue fragments formed multicellular spheroids. Following 5 days of culture, individual spheroids (150 to 500 μm in diameter) from 12 different patients were transferred with a sterile Pasteur pipette from the culture flasks into 16-mm agar-coated multiwell dishes with 2 ml growth medium. Spheroid size was estimated during a period of 20 days, using an inverted microscope with a calibrated reticle in the eyepiece. All further experiments, including proteomics were performed after 10 days of culture.

### Electron microscopy

Five spheroids from three tumors were collected, in each case, for transmission electron microscopy (TEM) or scanning electron microscopy (SEM) study. Spheroids were fixed for 3 hours in 2% glutaraldehyde in 0.1 M sucrose-adjusted cacodylate buffer (pH 7.4; 300 ± 10 mOsmol). Post fixation was performed for 1 hour in 1% OsO_4_ using the same buffer. The specimens were dehydrated with ethanol in concentrations increasing to 100%.

For TEM, embedding in Epon 812 was performed by using graded additions of Epon-propylenoxide mixtures. The final polymerization was carried out at 60°C for 3 days. Sections (60 nm thick) were cut with a diamond knife on a Reichert Ultracut E ultramicrotome (Leica, Buffallo, NY), collected on 200-mesh copper grids, and examined in a Jeol 1230 transmission electron microscope (Jeol, Tokyo, Japan) operated at 80 kV.

For SEM, the specimens were critical-point dried with CO_2_. They were then carefully mounted on stubs using tape and silver conductive paint. The specimens were coated with gold in a vacuum evaporator and examined with a Jeol JSM -7400 F Scanning Electron Microscope (Jeol, Tokyo, Japan).

### Light microscopy and immunohistochemistry

Part of collected material (Table 1) was routinely fixed for 48–72 hours (tumor tissue) or 12–24 hours (spheroids) in 4% formaldehyde solution, and embedded in paraffin. 5-μm- sections were hematoxylin and eosin (H&E) stained for light microscopical investigation.

For immunohistochemical (IHC) analyses, all blocking and staining materials were from Dako (Glostrup, Denmark) unless stated otherwise. Paraffin sections were dewaxed and rehydrated. The slides were heated in a microwave oven for 2 min at 1000 W, then 18 min at 200 W, in citrate buffer for antigen retrieval. Endogenous peroxidase activity was blocked with peroxidase block for 10 min. After washing with Tris-buffered saline (TBS) for 3 × 3 min, protein block was applied for 20 min, followed by primary antibodies for 45 min in a humid chamber at room temperature and sections were washed for 3 × 3 min with TBS. Envision system was used for visualization of antibody binding, with 3,3’-diaminobenzidine (DAB) as a chromogen. All sections were then counterstained with haematoxylin and mounted with Entellan mounting medium (Merck, Darmstadt, Germany).

The following antibodies and dilutions were used: anti-cytokeratin 20, 1:20 (Genetex, San Antonio, TX, USA), anti-vimentin, 1:100 (Dako, Glostrup, Denmark), anti-CD68, 1:50 (Dako, Glostrup, Denmark), anti-podoplanin, 1:100 (Acris, Hiddenhausen, Germany), Ki-67, 1:100, anti-PARP1, 1:100 (Bethyl, Antwerpen, Belgium), anti-Rac1, 1:50 (Chemicon, Overijse, Belgium), anti-β-catenin, 1:200 (Chemicon, Overijse, Belgium), anti-CD44 and anti-CD24, 1:50 (BD Biosciences, San Jose, CA, USA), anti-LAMA4 and anti-CDH1, 1:50 (Sigma Aldrich, Taufkirchen, Germany).

### Label-free proteomics by GeLC-MS/MS and spectral counting

#### Tissue lysis and gel electrophoresis

Three pairs of CRC biopsy samples and corresponding spheroid samples (uniformly 10 days in culture) were lysed in lysis buffer (per 100 mg tissue, one ml buffer containing 7 M urea, 2 M thiourea, 4% (w/v) CHAPS, and 10 μl/ml protease inhibitor mix (Amersham Biosciences, Piscataway, NJ, USA)). Thirty μg protein of each sample was separated in parallel on a 10% SDS-PAGE gel. The gel was fixed and stained with Coomassie Brilliant Blue, and each gel lane was sliced in 16 pieces (see Additional file 2: Figure S2). Proteins in the gel pieces were in-gel digested, and tryptic peptides extracted, as described elsewhere [92].

#### NanoLC-MSMS

The nanoLC-MS/MS workflow has been described before [45]. In brief, tryptic peptide fractions were injected on a nano-LC system (Ultimate 3000, Dionex LC-Packings, Amsterdam, The Netherlands) equipped with a 20 cm × 75 μm i.d.fused silica column custom packed with 3 μm 120 Å ReproSil Pur C18 aqua (Dr. Maisch, GMBH, Ammerbuch-Entringen, Germany). After injection, peptides were trapped at 30 μL/min on a 5 mm × 300 μm i.d. Pepmap C18 cartridge (Dionex LCPackings, Amsterdam, The Netherlands) at 2% buffer B (buffer A, 0.05% formic acid in MQ; buffer B, 80% ACN and 0.05% formic acid in MQ) and separated at 300 nL/min with a 10-40% buffer B gradient in 60 min.. Eluting peptides were ionized in a Nanomate Triversa Chip-based nanospray source using a Triversa LC coupler (Advion, Ithaca, NJ), and MS and MS/MS spectra were recorded on-line using an LTQ-FTICR mass spectrometer (ThermoFisher, Bremen, Germany).

#### Data analysis

Searching of MS and MS/MS data against version 3.31 of the human IPI (International Protein Index) database (67 511 entries), and spectral count analysis for relative quantification were performed as detailed before [45]. At the peptide level, only peptide identifications with a probability of >95% were considered, and at the protein level, only protein identifications with a probability of >99% and with at least two peptides identified in at least one of the samples were retained. Differential analysis of proteins in CRC tissue lysates versus corresponding spheroid lysates was performed by a pair-wise comparison of normalized spectral counts [72] where proteins with an FC >1.5 in each of the 3 pairs were subjected to further in silico analysis by protein network analysis and gene ontology mining (see below).

### Bioinformatics analysis

#### Identifier mapping

Using the human IPI identifiers of identified proteins, gene symbols were checked/retrieved using data from the European Bioinformatics Institute (EBI) IPI webpage (ftp://ftp.ebi.ac.uk/pub/databases/IPI) or, if the IPI identifier was no longer available in that repository, from the EBI Sequence Retrieval System (SRS) webpage (Present alternatives are suggested at http://srs.ebi.ac.uk) (neither of the two databases are no longer maintained) combined with a standard protein BLAST (blastp) search on the National Center for Biotechnology Information (NCBI) website (http://blast.ncbi.nlm.nih.gov). Subsequently, all gene symbols were mapped to official gene symbols using data from the HUGO Gene Nomenclature Committee (HGNC) website (http://www.genenames.org)

#### Analysis of protein-protein interaction networks

Lists of gene symbols associated with proteins that were up- or down-regulated, were analyzed with the Search Tool for the Retrieval of Interacting Genes/Proteins (STRING; version 8.3, http://string.embl.de). Protein-protein interaction (PPI) data were retrieved with default settings, allowing for experimentally verified as well as predicted PPIs. The results were exported as a text file and imported in the Cytoscape network analysis and visualization tool (version 2.8.1; http://www.cytoscape.org) [48]). Nodes were colored according to FC, and sized according to the number of edges. In the case of up-regulated proteins, proteins associated with acute phase reactions coagulation, or the complement cascade, as indicated by a preliminary gene ontology analysis (see below), were removed. These proteins are listed in Additional file 4: Table S1.

#### Gene ontology mining

Complete networks or subclusters were analyzed within Cytoscape, using the BINGO plug-in (version 2.44; http://apps.cytoscape.org/apps/bingo; [49]). Default settings were used (hypergeometric testing, Benjamini and Hochberg False Discovery Rate (FDR) correction, *P* < 0.05), and the whole data set was used as a reference set.

## Competing interests

The authors’ declare that they have no competing interests.

## Authors’ contributions

UR, SPN and CRJ have made substantial contributions to conception and design of the study, analysis and interpretation of data, have been involved in drafting the manuscript, revising it critically for important intellectual content and have given final approval of the version to be published. UR, JCK, SP, SB, FF, ES, TVP were involved in acquisition of data, analysis and interpretation of data and in drafting the manuscript and revising it critically. KS, RM were involved in critical revision of the study and have given final approval of the version to be published. All authors read and approved the final manuscript.

## Supplementary Material

Additional file 1 **Figure 1A.** Average spheroid size depending on the time in culture. **Figure 1B.** Proliferation Index of colorectal tumors and corresponding spheroids. Click here for file

Additional file 2: Figure S2 SDS PAGE gel image showing separation pattern of tumor and corresponding spheroid proteins. Click here for file

Additional file 3: Figure S3 Venn diagrams showing the overlap of protein identifications accross biological samples. Click here for file

Additional file 4: Table S1 Up-regulated nodes deleted from Figure 5 (Acute phase/coaggulation/complement cascade associated). Click here for file

Additional file 5: Table S2 Comparison of parental CRC tissue (CRC) vs derived spheroids (SPH) (paired samples) - All Data. Click here for file

Additional file 6: Table S3A-D Gene Ontology representation by Cytoscape and Bingo analyses. Click here for file

Additional file 7: Table S4 Comparison of parental CRC tissue (CRC) vs derived spheroids (SPH) (paired samples) - Proteins exhibiting Fold Change > 1.5-fold in at least 2 out of 3 CRC/SPH pairs. Click here for file
