# Supplementary figures and images for: Colorectal cancer derived organotypic spheroids maintain essential tissue characteristics but adapt their metabolism in culture
Source: Proteome Sci. 2014 Jul 11;12:39. doi: 10.1186/1477-5956-12-39 (PMC4114130; doi:10.1186/1477-5956-12-39)

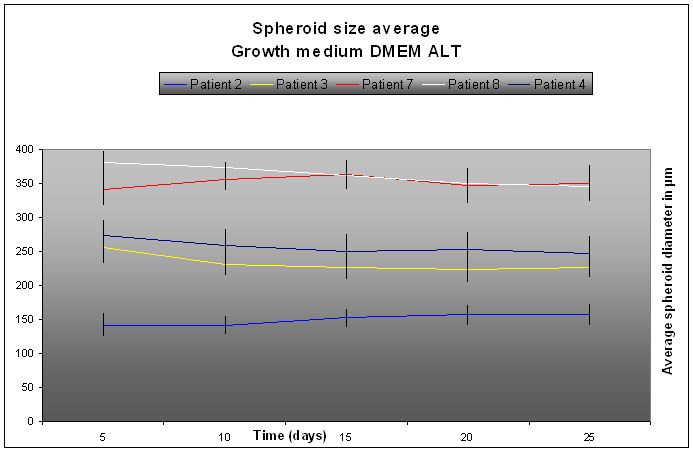

Supplement: Additional file 1 — Figure 1A. Average spheroid size depending on the time in culture. Figure 1B. Proliferation Index of colorectal tumors and corresponding spheroids. [file 1477-5956-12-39-S1.zip › 2870410651112261_add1a.tiff]

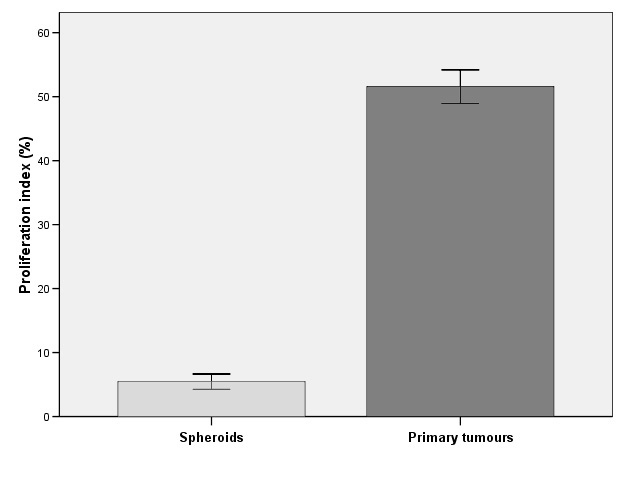

Supplement: Additional file 1 — Figure 1A. Average spheroid size depending on the time in culture. Figure 1B. Proliferation Index of colorectal tumors and corresponding spheroids. [file 1477-5956-12-39-S1.zip › 2870410651112261_add1b.tiff]

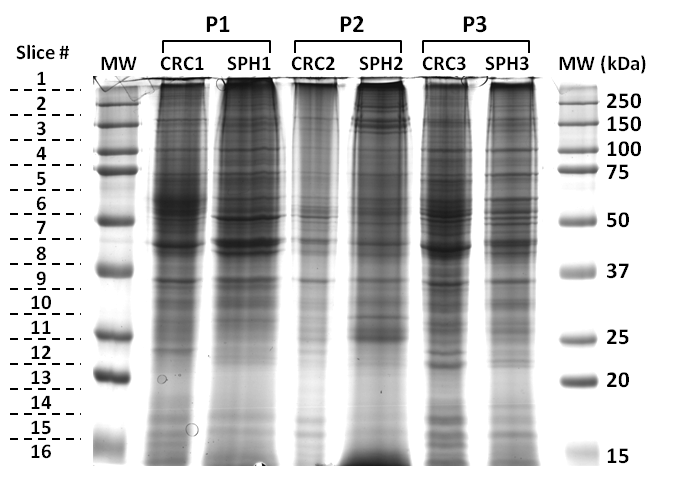

Supplement: Additional file 2: Figure S2 — SDS PAGE gel image showing separation pattern of tumor and corresponding spheroid proteins. [file 1477-5956-12-39-S2.tiff]

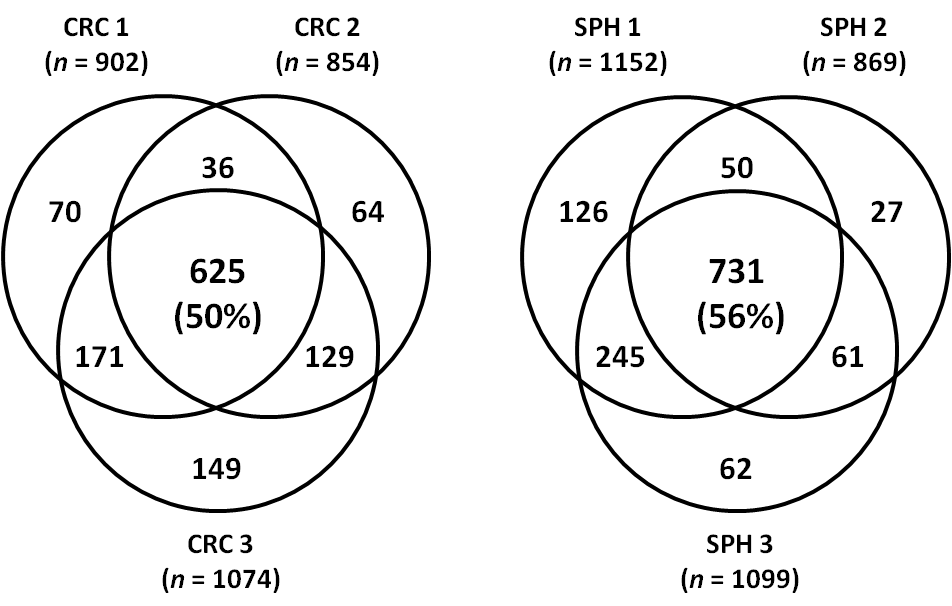

Supplement: Additional file 3: Figure S3 — Venn diagrams showing the overlap of protein identifications accross biological samples. [file 1477-5956-12-39-S3.tiff]
